# Deep-Sea Actinobacteria Mitigate Salinity Stress in Tomato Seedlings and Their Biosafety Testing

**DOI:** 10.3390/plants10081687

**Published:** 2021-08-17

**Authors:** Pharada Rangseekaew, Adoración Barros-Rodríguez, Wasu Pathom-aree, Maximino Manzanera

**Affiliations:** 1 Doctor of Philosophy Program in Applied Microbiology (International Program) in Faculty of Science, Chiang Mai University, Chiang Mai 50200, Thailand; Pharada_ra@cmu.ac.th; 2 Graduate School, Chiang Mai University, Chiang Mai 50200, Thailand; 3 Department of Microbiology, Institute for Water Research, University of Granada, 18071 Granada, Spain; dorysbr@correo.ugr.es (A.B.-R.); manzanera@ugr.es (M.M.); 4 Research Center of Microbial Diversity and Sustainable Utilization, Department of Biology, Faculty of Science, Chiang Mai University, Chiang Mai 50200, Thailand

**Keywords:** biosafety, *Dermacoccus*, marine actinobacteria, plant growth promotion, salt stress, sustainable agriculture

## Abstract

Soil salinity is an enormous problem affecting global agricultural productivity. Deep-sea actinobacteria are interesting due to their salt tolerance mechanisms. In the present study, we aim to determine the ability of deep-sea *Dermacoccus* (*D. barathri* MT2.1^T^ and *D. profundi* MT2.2^T^) to promote tomato seedlings under 150 mM NaCl compared with the terrestrial strain *D. nishinomiyaensis* DSM20448^T^. All strains exhibit in vitro plant growth-promoting traits of indole-3-acetic acid production, phosphate solubilization, and siderophore production. Tomato seedlings inoculated with *D. barathri* MT2.1^T^ showed higher growth parameters (shoot and root length, dry weight, and chlorophyll content) than non-inoculated tomato and the terrestrial strain under 150 mM NaCl. In addition, hydrogen peroxide (H_2_O_2_) in leaves of tomatoes inoculated with deep-sea *Dermacoccus* was lower than the control seedlings. This observation suggested that deep-sea *Dermacoccus* mitigated salt stress by reducing oxidative stress caused by hydrogen peroxide. *D. barathri* MT2.1^T^ showed no harmful effects on *Caenorhabditis elegans*, *Daphnia magna*, *Eisenia foetida*, and *Escherichia coli* MC4100 in biosafety tests. This evidence suggests that *D. barathri* MT2.1^T^ would be safe for use in the environment. Our results highlight the potential of deep-sea *Dermacoccus* as a plant growth promoter for tomatoes under salinity stress.

## 1. Introduction

Marine ecosystems cover more than 70% of the surface of the Earth, with most parts still under-explored [1]. The deep-sea is still poorly investigated in terms of microbial diversity due to the difficulty in obtaining the samples. Nevertheless, actinobacteria are widely distributed in deep-sea environments [2,3,4]. These marine actinobacteria are considered an excellent source of bioactive compounds. The adaptation of deep-sea microorganisms is interesting from an academic perspective and for their potential applications in biotechnology. The deep-sea environment is generally characterized by high pressure, low temperature, lack of light with varying concentrations of oxygen and salinity [2,5]. Deep-sea actinobacteria have to adapt or develop unique abilities to withstand salinity and pressure [6] or piezotolerant properties [7] to survive under these extreme conditions. With the adaptive ability to survive under extreme environments, especially salinity and osmotic stress, these deep-sea actinobacteria are likely to be useful as biostimulants to mitigate salinity stress for plant growth.

Salinity stress is considered a major problem causing a massive reduction in agricultural production worldwide. The total area of global cultivated land is 1.5 × 10^9^ hectares; among these, 23% are saline soil and 37% sodic soil, between 25% and 30% of irrigated lands are also affected by salt [8]. Salinization impact on irrigated land is estimated to cost at least 12 billion US$ in the annual global income [9,10]. High salinity damages plant growth and development mainly by ionic and osmotic stress [11,12]. Ionic stress leads to an excessive influx of sodium ions, resulting in an overall efflux of potassium ions [12,13,14,15]. Osmotic stress causes the accumulation of salts in the soil surrounding plant roots and leads to low water potential in soils. Hence, plants suffer from water and nutrients uptake interruption [6,16,17,18,19]. Salinity stress alters several plant morphological traits, such as a reduction in the number of leaves, plant size, roots length, and flower appearance [20,21,22,23]. Moreover, salinity induces the production of reactive oxygen species (ROS) causing a direct injury to plant tissues [16,24,25,26] and secondary DNA damages, such as base loss, DNA-protein crosslinks, and double-stranded DNA breakage [12,27]. In addition, the gradual accumulation of salts in plant parts damages cell membrane integrity and decreases membrane stability index [13,24,28,29]. Salinity also decreases photosynthesis through chlorophyll content reduction [30,31,32]. These biochemical and physiological damages are reflected in the poor growth of plants and reduce their overall productivity [16,31,33].

Several options are available to solve salinity stress problems ranging from conventional plant breeding programs to the development of salt-tolerant crop plants by genetic engineering [34]. However, these conventional methods are time-consuming, labor-intensive, rely on well-characterized germplasms, and may introduce undesirable traits along with the selected one [30,35]. The use of safe plant growth-promoting bacteria (PGPB) is an alternative option for drought and salinity stress mitigation [36,37,38,39,40]. Actinobacteria are considered a promising group of PGPB which offer an alternative environment-friendly way to mitigate the negative effects of salinity stress. Actinobacteria were reported to be potential plant growth promoters under normal and stress conditions. They act via mechanisms, such as phytohormone production, siderophore production, and phosphate solubilization [41,42,43,44]. Actinobacteria are also considered powerful biocontrol agents due to their antibiotic and lytic enzyme production to inhibit plant pathogens [41,43,45,46]. *Streptomyces* are one of the largest genera of Actinobacteria, with over 500 species [47]. These filamentous bacteria are well accepted as the most significant producer of bioactive compounds with extensive use in industrial applications [48,49,50,51]. *Streptomyces* are also widely used in agriculture as a supplement to enhance plant growth and inhibit plant pathogens—for instance, *Streptomyces* sp. KLBMP S0051 isolated from coastal salt marsh rhizosphere soils in China could produce indole-3-acetic acid (IAA) and promote seed germination of wheat variety Bainong AK58 and enhance seedling growth under salt stress [52]. *Streptomyces* spp. were also reported to promote growth in other plants, such as rice [53] and tomato [32,54]. Non-*Streptomyces* are also a good producer of bioactive compounds, with at least 2500 compounds reported [50]. However, research on non-*Streptomyces* actinobacteria to promote plant growth under abiotic stress is still limited. *Micrococcus* from forest soil in India showed plant growth-promoting traits in vitro and promoted cowpea growth [55]. Other non-*Streptomyces* genera with plant growth-promoting potential include *Curtobacterium flaccumfaciens*, which mitigated barley growth under salinity [56], *Microbacterium* sp. 3J1, which increased pepper growth under desiccation [57] and *Arthrobacter* strains MS-1 and MS-2, which promote the growth of alfalfa under non-saline and 100 mM NaCl [40].

The discovery of extremophilic actinobacteria is interesting because of their adaptations to extreme environments leading to the production of unique bioactive compounds [52]. Actinobacteria have been found in various marine environments, including mangroves [58,59,60] and deep-sea sediments [2,3,61]. Most plant growth-promoting actinobacteria are isolated from non-saline environments. Information on actinobacteria from marine habitats with plant growth promotion is still scarce but beginning to attract attention. For example, *Nocardiopsis yanglingensis*, *Streptomyces jiujiangensis*, *S. psammoticus*, and *Pseudonocardia oroxyli* from mangrove areas in Thailand can produce IAA, siderophore, and phosphate solubilization and promote growth of rice (*Oryza sativa*) seedlings under normal and salinity conditions up to 200 mM NaCl [60]. Similarly, mangrove-associated *S. iranensis* S2-SC16 with IAA, siderophore producing, and phosphate solubilization abilities promote Thai jasmine rice (*O. sativa* KDML105) seedlings. This isolate also showed potential as a biocontrol agent for rice Bakanae disease caused by *Fusarium fujikuroi* [53]. In addition, *Saccharomonospora* sp. from the marine soil of Morocco have shown in vitro that both solubilize phosphorous and produce IAA and siderophores [62]. Recently, coral-associated actinobacteria were reported to alleviate salt stress during the germination and photosynthesis of tobacco plants [63]. However, deep-sea actinobacteria have never been explored in terms of their benefits to plants. Members of the genus *Dermacoccus* had previously been isolated from the Challenger Deep of the Mariana Trench in the western Pacific Ocean [3,7,64]. We suggest that the ability of these deep-sea actinobacteria to survive under constant high-salinity and osmotic stress would be useful to help plant growth under salt stress. Hence, this research aims to determine the ability of two deep-sea actinobacteria, *D. barathri* MT2.1^T^ and *D. profundi* MT2.2^T^ which could tolerate up to 10% NaCl to promote tomato growth (*Solanum lycopersicum*) under salinity stress. A type species of the genus, *D. nishinomiyaensis* DSM20448^T^ originated from a non-marine environment was included for comparison. The safety of these strains was also evaluated to ensure no negative effect on organisms in the environments according to the Environmental and human safety index (EHSI) [65].

## 2. Results

### 2.1. Plant Growth-Promoting Properties In Vitro

All 3 strains of actinobacteria could produce IAA in tryptophan-added culture broth in the range of 6.18–16.64 µg mL^−1^ (Table 1). *D. nishinomiyaensis* DSM20448^T^ produced the highest concentration of IAA of 16.64 µg ml^−1^ in the absence of salt stress. Under salt stress at NaCl concentrations of 150, 300, and 450 mM, IAA production of *D. barathri* MT2.1^T^ showed no significant difference between culture broth with or without NaCl. Interestingly, *D. nishinomiyaensis* DSM20448^T^ and *D. profundi* MT2.2^T^ showed no significant difference in IAA production between 0 mM and 150 mM NaCl. However, IAA production of both strains increased at a higher NaCl level (300 and 450 mM NaCl). All actinobacteria produced siderophores on CAS agar. In addition, all strains grew in King’s B broth and produced a varying quantity of siderophores (Table 2). Hydroxamate type siderophore was produced in a significantly higher quantity than catecholate type. The highest production of hydroxamate and catecholate type siderophores was observed in *D. barathri* MT2.1^T^ at 240.00 µmol mL^−1^ and *D. nishinomiyaensis* DSM20448^T^ at 22.63 µmol mL^−1^, respectively. All strains produced a clear zone of tri-calcium phosphate solubilization on PVK agar, as shown in Table 2. Deep-sea *Dermacoccus* strains released higher phosphorus in the culture broth. The pH of the culture broth was reduced from 7 to below 5 (4.62–4.78). All *Dermacoccus* strains showed low growth on ACC containing minimal medium compared to minimal medium supplemented with ammonium sulfate (positive control). Therefore, they were not considered ACC deaminase-producing strains (Figure S2).

### 2.2. Enhancement of Tomato Seedlings Growth without Salt Stress

**Figure 1:**
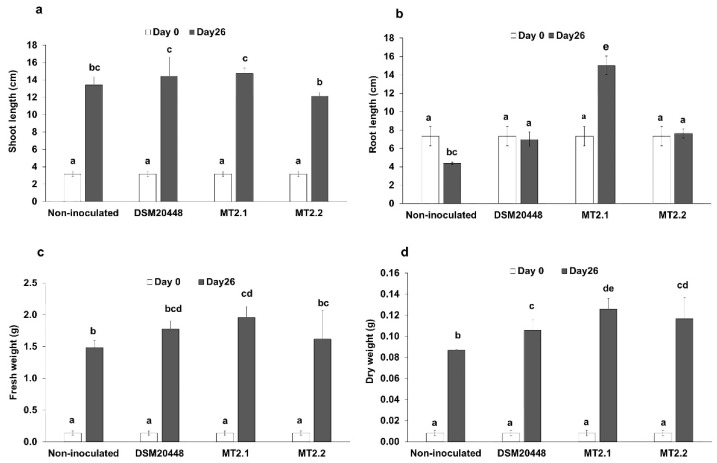
Deep-sea actinobacteria promote tomato seedlings’ growth without salt stress. (**a**) shoot length; (**b**) root length; (**c**) fresh weight; (**d**) dry weight. Data represent the mean values of three replicates. Different letters (a,b,c,e,de,cd,bc,bcd) indicate a significant difference according to Duncan at *p* < 0.05.

*Dermacoccus* strains were applied to tomato seedlings to evaluate their growth-promoting effects (Figure 1a–d). The inoculation of *Dermacoccus* strains increased the growth of tomato seedlings compared to non-inoculated control in terms of dry weight (Figure 1d) and root length (Figure 1b). *D. barathri* MT2.1^T^ significantly promoted the root length in comparison to the non-inoculated control or other *Dermacoccus* strains (*p* < 0.05).

### 2.3. Enhancement of Tomato Seedlings Growth under Salt Stress

**Figure 2:**
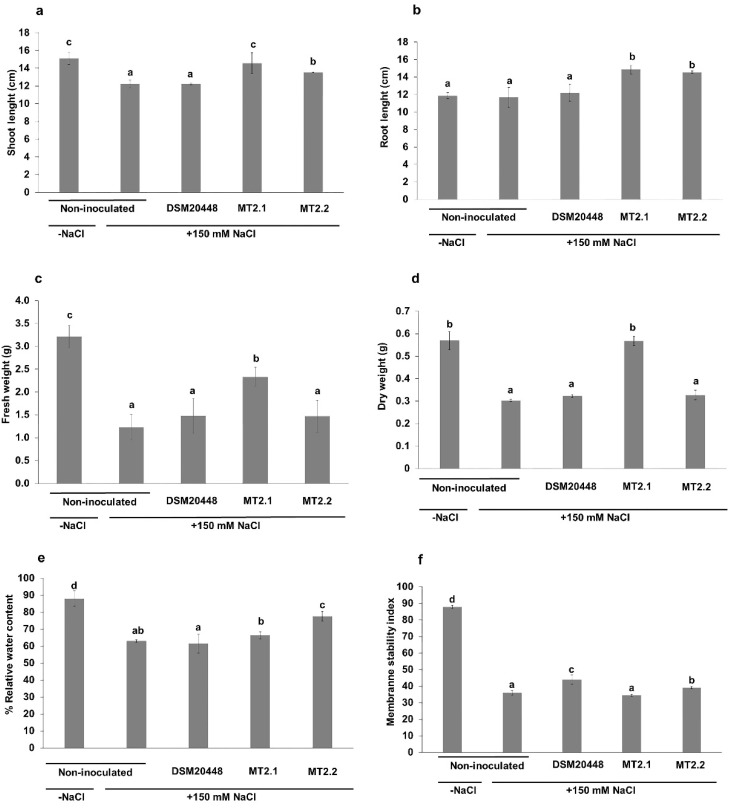
Effect of deep-sea actinobacteria on physiological properties of tomato seedlings under salinity stress (150 mM NaCl). (**a**) shoot length; (**b**) root length; (**c**) fresh weight; (**d**) dry weight; (**e**) percentage of relative water content (% RWC); (**f**) membrane stability index (MSI). Data represent the mean values of three replicates ± SD. Different letters (a,b,c,d) indicate a significant difference according to Duncan at *p* < 0.05. −NaCl means non-stressed condition; +150 mM NaCl means salinity stress condition.

**Figure 3:**
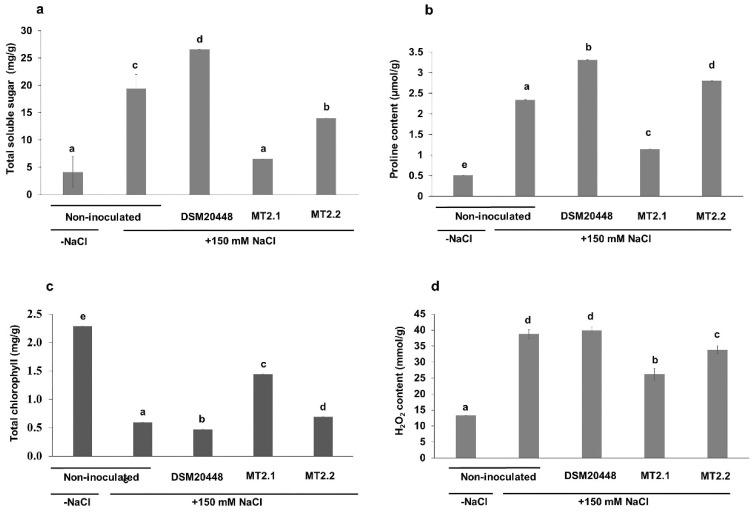
Effect of deep-sea actinobacteria on biochemical properties of tomato seedlings under salinity stress (150 mM NaCl). (**a**) total soluble sugar; (**b**) proline content; (**c**) total chlorophyll; (**d**) content of hydrogen peroxide. Data represent the mean values of three replicates ± SD. Different letters (a,b,c,d) indicate a significant difference according to Duncan at *p* < 0.05. −NaCl means non-stressed condition; +150 mM NaCl means salinity stress condition.

The effects of *Dermacoccus* strains on tomato seedlings under salinity stress at 150 mM NaCl are shown in Figure 2 and Figure 3. In general, tomatoes inoculated with *Dermacoccus* strains showed better growth than non-inoculated seedlings. At the end of the experiment (14 days), tomato inoculated with *D. barathri* MT2.1^T^ showed shoot length (Figure 2a), root length (Figure 2b), fresh weight (Figure 2c), and dry weight (Figure 2d) significantly higher than non-inoculated tomato under stress. Moreover, tomato inoculated with *D. nishinomiyaensis* DSM20448^T^ showed no significant differences in shoot length, root length, dry weight, and fresh weight compared with non-inoculated tomato seedlings under salt stress (Figure 2a–d).

Furthermore, when comparing tomato inoculated with deep-sea strains (*D. barathri* MT2.1^T^ and *D. profundi* MT2.2^T^) and a terrestrial strain (*D. nishinomiyaensis* DSM20448^T^), deep-sea strains showed better shoot and root length in tomato seedling under salt stress. In particular, *D. barathri* MT2.1^T^ showed the highest values of shoot length, root length, fresh weight, and dry weight (Figure 2a–d). In addition, similar shoot length and dry weight were observed between tomato inoculated with *D. barathri* MT2.1^T^ and control tomato without salt stress (non-inoculated) (Figure 2a,d).

The highest percentage of RWC was observed in non-stressed tomato, while tomatoes inoculated with deep-sea strains of *D. barathri* MT2.1^T^ (66.39%) and *D. profundi* MT2.2^T^ (77.62%) showed a higher RWC than inoculated tomato seedlings with *D. nishinomiyaensis* DSM20448^T^ (61.59%) under stress conditions (Figure 2e). However, tomato-inoculated with *D. barathri* MT2.1^T^ and *D. nishinomiyaensis* DSM20448^T^ showed no significant difference in RWC compared with non-inoculated tomato (62.99%) under salt stress (Figure 2e). MSI in all tomatoes inoculated with actinobacteria under salinity stress was significantly higher than in non-inoculated tomatoes with stress except for *D. barathri* MT2.1^T^ (Figure 2f).

The effect of salt stress on the morphology of tomato seedlings was also recorded (Figure S1). At the end of the experiment, non-inoculated tomato seedlings under stress conditions showed curly and wilting leaves and faded leaf color (Figure S1b). However, tomato seedlings inoculated with deep-sea *Dermacoccus* strains showed better overall growth (Figure S1c–e). Leaves on the upper part of tomato seedling-inoculated with deep-sea *Dermacoccus* strains were still healthy compared to non-inoculated with stress tomato seedlings. Moreover, the root architecture of inoculated seedlings was improved as observed from an increased length of the main root and the number of lateral root branching.

#### Biochemical Analysis

The biochemical response of tomato seedlings with inoculated actinobacteria in mitigating salinity stress was determined in proline content, TSS, hydrogen peroxide content, and chlorophyll content (Figure 3a–d). For TSS and proline contents, the inoculation of tomato seedlings with actinobacteria resulted in a varied response in a species-dependent manner. For example, tomato seedlings inoculated with *D. nishinomiyaensis* DSM20448^T^ showed higher TSS and proline content than the non-inoculated tomato under salt stress. However, there is a pronounced and significant decrease of TSS in tomato seedlings inoculated with both the deep-sea strains and of proline with *D. barathri* MT2.1^T^ than non-inoculated tomato under salt stress (Figure 3a,b). Interestingly, tomatoes inoculated with *D. barathri* MT2.1^T^ showed a similar TSS level to the non-inoculated tomato plants under non-stressed conditions (Figure 3a). Higher proline accumulation was observed in all stressed tomato plants with and without inoculated actinobacteria (Figure 3b). Total chlorophyll content in deep-sea *Dermacoccus* inoculated tomato seedlings was significantly higher than non-inoculated tomatoes under stressing conditions. Plants inoculated with *D. nishinomiyaensis* DSM20448^T^ presented the lowest chlorophyll content, whereas the highest content was observed in non-inoculated plants in the absence of salt stress (Figure 3c). The hydrogen peroxide content (H_2_O_2_) in tomato seedlings inoculated with the deep-sea *Dermacoccus* was lower than in the non-inoculated tomato and tomato inoculated with *D. nishinomiyaensis* DSM20448^T^, both of them under salty stress (Figure 3d). In addition, non-inoculated tomato plants in non-stressing conditions showed the lowest hydrogen peroxide value compared to tomato plants under salt stress regardless of the addition of actinobacteria (Figure 3d).

### 2.4. Biosafety Tests for Actinobacteria

#### 2.4.1. Pathogenicity Bioassay Based on *Caenorhabditis elegans*

**Figure 4:**
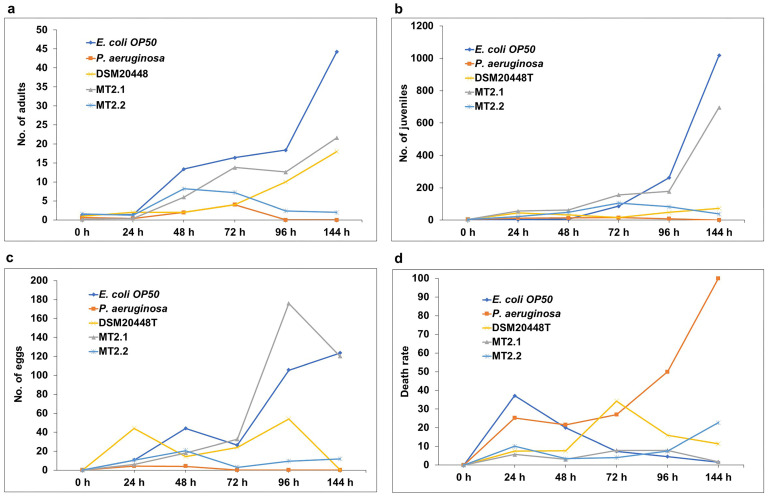
Pathogenicity bioassay based on *Caenorhabditis elegans*. Time course of changes in the number of (**a**) adults; (**b**) juveniles; (**c**) eggs; (**d**) death rate. Data represent the mean values of three replicates ± SD. Different letters indicate a significant difference according to Duncan at *p* < 0.05.

Pathogenicity bioassay was performed to evaluate the effect of actinobacteria on the number of adults, juveniles, eggs, and dead *C. elegans* (Figure 4a–d). When fed with *P. aeruginosa* PA14 (as pathogenic strain), the lowest number of adults, juveniles, and eggs were found, and all nematodes died after 96 h. The highest number of adults, juveniles, and eggs were found when fed with *E. coli* OP50 (as a non-pathogenic strain) at 144 h. Nematodes fed with *D. barathri* MT2.1^T^ showed a 51% reduction in the number of adults and 31% of juveniles compared to control (*E. coli* OP50). For *D. nishinomiyaensis* DSM20448^T^, the number of adults and juveniles decreased by 59% and 92%. The highest reduction (96%) in the number of adults and juveniles was observed in nematodes fed with *D. profundi* MT2.2^T^. The number of eggs at 144 h was similar between *E. coli* OP50 (123 eggs) and *D. barathri* MT2.1^T^ (120 eggs). However, nematodes fed *with D. nishinomiyaensis* DSM20448^T^ did not hatch an egg at 144 h, and *D. profundi* MT2.2^T^ showed the lowest number of eggs (12 eggs). For the percentage of death at 144h, *P. aeruginosa* PA14 showed the highest death (100%), followed by *D. profundi* MT2.2^T^ (23%), *D. nishinomiyaensis* DSM20448^T^ (11%), *D. barathri* MT2.1^T^ (1.8%), and *E. coli* OP50 (1.6%) (Figure 4d).

#### 2.4.2. *Escherichia coli* MC4100 Sensitivity

**Figure 5:**
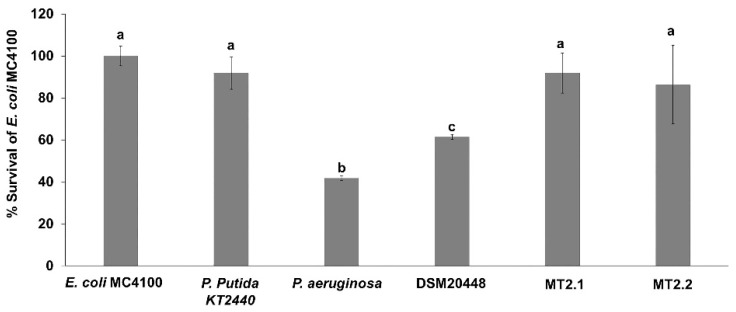
*Escherichia coli* MC4100 sensitivity. Data represent the mean values of three replicates ± SD. Different letters (a,b,c) indicate a significant difference according to Duncan at *p* < 0.05.

Some actinobacteria can produce and release secondary metabolites to the environment affecting the microbial community. Therefore, we investigated the effects of the supernatant of actinobacteria on *E. coli* MC4100 cells (Figure 5). Survival rate (%) of *E. coli* MC4100 cells when exposed to cell-free supernatant from P. putida KT2440, *D. barathri* MT2.1^T^, and *D. profundi* MT2.2^T^ were 91.9%, 91.9%, and 86.5%, respectively. However, more than 40% reduction in *E. coli* MC4100 cells was found when exposed to supernatants of *P. aeruginosa* PA14 (41.9%) and *D. nishinomiyaensis* DSM20448^T^ (61.5%).

#### 2.4.3. Ecotoxicity Test in Earthworms (*Eisenia foetida*)

**Figure 6:**
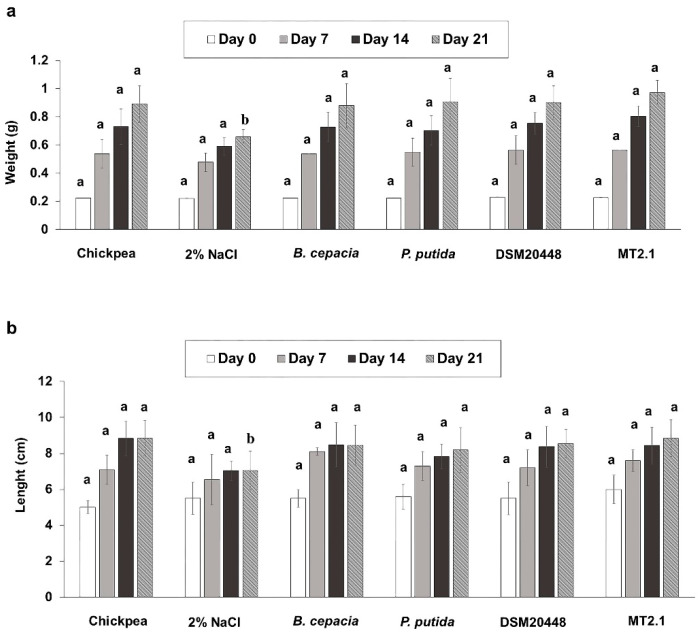
Ecotoxicity tests in earthworms (*Eisenia foetida*). Time course of changes in (**a**) weight; (**b**) length of *Eisenia foetida* after incubation with *B. cepacia* CC-A174 or *P. putida* KT2440 or *D. nishinomiyaensis* DSM20448^T^ or *D. barathri* MT2.1^T^. Data represent the mean values of three replicates ± SD. Different letters (a,b) indicate a significant difference in weight and length of earthworms at different cultivation days according to Duncan at *p* < 0.05.

The effects of actinobacteria on annelids in soil were investigated using earthworms (*E. foetida*). The development of earthworms was determined by measuring the weight, length, and number of ootheca (Figure 6). At day 21, a similar pattern of increasing weight in earthworms fed with only chickpea (positive control, 75%) or chickpea supplemented with different bacteria: *P. putida* KT2440 (safe strain; 66%), *B. cepacia* CC-A174 (pathogenic strain; 75%), *D. nishinomiyaensis* DSM20448^T^ (75%) and *D. barathri* MT2.1^T^ (77%) was found. A similar increasing trend of earthworm length was also observed when fed with bacteria-supplemented chickpea (46–55%) compared with non-supplemented chickpea. The addition of 2% NaCl resulted in the lowest value for both the weight and length of earthworms. For the number of eggs, the addition of *P. putida* KT2440 (12.5 eggs/earthworm) showed the highest number of ootheca, followed by *D. nishinomiyaensis* DSM20448^T^ (10.4 eggs/earthworm), chickpea (10.3 eggs/earthworm), *D. barathri* MT2.1^T^ (10 eggs/earthworm) and *B. cepacia* CC-A174 (9.6 eggs/earthworm). The lowest number of ootheca was observed in chickpea supplemented with 2% (*w/w*) NaCl (4.8 eggs/earthworm) (data not shown).

#### 2.4.4. *Daphnia magna* Toxicity Bioassay

*Daphnia magna* was used to study the impact of microbial metabolites on the aquatic ecosystem. TSB was used for suspension preparation and standard freshwater as control. Standard freshwater showed no effect on *D. magna*. The addition of spent media from *B. cepacia* CC-A174 had the most adverse effect on *D. magna* survival as all Daphnia were dead at the lowest concentration tested (3.125%). The EC50 value of *D. barathri* MT2.1^T^ was 6.25% at 24 h. However, the addition of spent media at this concentration from the culture of both *D. nishinomiyaensis* DSM20448^T^ and *P. putida* KT2440 had no adverse effect on *D. magna* (Data not shown).

## 3. Discussion

### 3.1. Plant Growth-Promoting Properties In Vitro

Indole-3-acetic acid (IAA) is a major auxin-type phytohormone that helps the plant grow and develop. It also contributes to the growth of young seedlings [26], embryo and fruit development, and especially root hair formation [66]. Many actinobacteria were reported to produce IAA, such as *Arthrobacter* sp., *Nocardioides* sp. [67], *Pseudonocardia* sp. [68], and *Streptomyces* spp. [68,69]. Marine actinobacteria were also able to produce IAA, e.g., *Streptomyces* spp. isolated from the Egyptian coastal area produced IAA in the range of 4.12–49.7 µg mL^−1^ [70]. *Saccharomonospora* sp. LNS-1 isolated from the largest lagoon in Morocco produces 49.46 µg mL^−1^ IAA in vitro [62]. In the present study, deep-sea *D. barathri* MT2.1^T^ and *D. profundi* MT2.2^T^ showed lower IAA production than a terrestrial strain of *D. nishinomiyaensis* DSM20448^T^ in tryptophan-added culture broth without NaCl. All strains still produced IAA under increased NaCl concentration up to 450 mM NaCl. An increasing NaCl concentration did not affect the IAA production of *D. barathri* MT2.1^T^. However, NaCl concentration seems to have both negative and positive effects on the IAA production of *D. nishinomiyaensis* DSM20448^T^ and *D. profundi* MT2.2^T^. At low NaCl concentration (150 mM NaCl), IAA production was reduced in both *D. nishinomiyaensis* DSM20448^T^ and *D. profundi* MT2.2^T^ (Table 1). On the contrary, IAA production was increased in both strains at higher NaCl concentrations (300 and 450 mM NaCl). A similar observation was found in *Streptomyces* isolate C isolated from Iranian wheat field; that is, the IAA production increased (2.4–4.7 µg mL^−1^) with an increasing NaCl concentration from 100–300 mM [71]. The production of IAA from marine actinobacteria is rarely reported. The only available information was from mangrove actinobacteria [53,60]. No attempt had been previously performed to investigate plant growth-promoting properties in deep-sea actinobacteria, including IAA production. However, the mechanism on how NaCl inserts a positive effect on IAA production remains unclear and needs additional research.

Siderophores are ferric iron (Fe^3+^) specific chelators, facilitating iron uptake under limiting iron [72,73]. Siderophores are produced by some microorganisms, including actinobacteria. Siderophores are known to promote plant growth via both direct (iron uptake) and indirect mechanisms (pathogen inhibition) [71,74]. Siderophores are classified into hydroxamate, catecholate, and carboxylate types. Interestingly, *Dermacoccus* spp. produced higher hydroxamate than catecholate type siderophore in King’s B broth. Phosphorus is one of the major macronutrients required for plant growth. Phosphorus in the soil is found in an insoluble form that cannot be used by plants. However, some microorganisms can solubilize phosphate and release phosphorus into the soil. In the present study, all strains solubilized tri-calcium phosphate in the culture broth. Liquid media was more suitable for determining phosphate solubilization by bacteria [75]. A drop in pH of culture broth to 4.62–4.78 was also observed. A reduction in the pH of the media from organic acid production is a known primary mechanism of phosphate solubilization in microorganisms, including actinobacteria [68,76]. Deep-sea *Dermacoccus* strain released phosphorus almost 4 times higher than their terrestrial neighbor, *D. nishinomiyaensis* DSM20448^T^. High phosphate solubilizing activity was also reported from members of genera *Streptomyces*, *Microbacterium*, *Angusibacter*, *Kocuria*, *Isoptericola*, and *Agromyces* isolated from sediment in Chorao Island, India [77].

The enzymatic activity of ACC deaminase results in the degradation of ACC, which is the precursor of ethylene in all higher plants [12]. Ethylene level in plants is increased under salinity stress, leading to series of abnormal morphologies, such as leaf yellowing, the senescence of various organs, the abscission of leaves, petals, and flowers, and premature death [12,78]. A lower level of ACC reduced ethylene synthesis resulting in lower stress levels in plants. However, all *Dermacoccus* strains showed no ACC deaminase activity. This observation suggested that these *Dermacoccus* strains used alternative mechanisms to alleviate salinity stress in tomato seedlings.

### 3.2. Promotion of Tomato Growth Using Dermacoccus Strains

Inoculation of *Dermacoccus* strains positively affected the growth of tomato seedlings under non-stressed conditions as seen from the dry weight and root length compared to non-inoculated seedlings. Although studies of marine actinobacteria to enhance plant growth are still limited, actinobacteria from other saline habitats were used as plant growth promotors. Only actinobacteria from mangrove in Thailand, which belong to the genera *Nocardiopsis*, *Pseudonocardia*, and *Streptomyces* were reported to promote the growth of rice seedlings without salt stress and up to 200 mM NaCl [60]. Similarly, *Streptomyces* sp. KLBMP S0051 from Chinese coastal salt marsh rhizosphere soils, promoted the growth of wheat seedlings with and without NaCl [52]. Recently, *Glutamicibacter halophytocola* KLBMP 5180 isolated from the root of Chinese coastal halophyte, increased the growth of tomato seedlings under the non-saline condition in terms of fresh weight, height, root length, number of fibrous roots, and total chlorophyll content [79]. It is evident from ours results that deep-sea *Dermacoccus* have no negative effect on tomato growth and were selected to test their ability to promote the growth of tomato seedlings under salinity stress.

Although the ecological roles of marine actinobacteria remain largely unknown and they need to be determined, actinobacteria from the deep-sea environments have to constantly tolerate salinity, high pressure, low temperature, and frequently low nutrient availability. It is not surprising that they have to evolve unique properties to survive under such extreme environments. The Mariana Trench, the deepest location on earth, is remarkably high in hydrostatic pressure and salinity [80]. Recently, genes related to stress response, especially those related to osmotic stress response, e.g., synthesis and accumulation of compatible solutes, and oxidative stress response, e.g., ROS scavenging enzymes, which may play an essential role in the evolution of marine actinobacteria to tolerate salinity stress were reported for *D. abyssi* MT1.1^T^ [81]. From this evidence, we designed the experiment to test the ability of two other deep-sea *Dermacoccus* from the Mariana Trench to mitigate salt stress on tomato growth.

Salt stress is one of the most important abiotic stress limiting agricultural productivity. Soil salinity causes an alteration in biochemical and physiological properties, such as ion toxicity, osmotic stress, mineral uptake, nutrient deficiency, and photosynthetic activity [14,71]. From our results, salinity stress at 150 mM NaCl clearly asserted a negative effect on tomato morphology (Figure S1b) as observed in the number of wither leaves, the reduced main root length, and the number of lateral root branching. A similar observation in tomato (*Solanum lycopersicum* L.) was reported by Karni et al. [82] who found that 100 mM NaCl reduced the length of the main root. However, an increase in the number of branching roots was also recorded. This inconsistency is not surprising because the lateral root formation can be less affected or might even be stimulated by the salinity stress [83].

In the present study, we evaluated the effects of deep-sea actinobacteria on tomato seedlings under salt stress (150 mM NaCl). As observed, deep-sea actinobacteria (*D. barathri* MT2.1^T^ and *D. profundi* MT2.2^T^) were able to improve tomato seedling appearance as tomatoes inoculated with these two strains showed higher shoot and root length than non-inoculated tomato plants under salt stress (Figure 2a,b). Similar results were stated by Tank et al. [84] who reported the ability of rhizobacteria isolated from tomato fields to increase tomato growth (shoot and root length, numbers of leaves, and lateral roots) under 2% NaCl. In addition, inoculation of tomatoes with *D. barathri* MT2.1^T^ resulted in a higher shoot length, root length, fresh and dry weight than non-inoculated tomato plants under stress conditions. Palaniyandi et al. [31] isolated *Streptomyces* PGPA39 from agricultural soils, which alleviated salt stress (180 mM NaCl) in Micro-Tom tomato. This strain improved shoot length, root length, and dry biomass of tomato due to IAA production. Production of IAA by plant growth-promoting rhizobacteria has been reported that helps in root formation and root architecture modification, resulting in an increased root area and nutrient uptake efficiency of plants under salinity stress [15]. It is interesting to note that tomato inoculated with a terrestrial strain of *D. nishinomiyaensis* DSM20448^T^ showed no significant difference in growth performance in terms of shoot length, root length, fresh and dry weight compared with non-inoculated tomato under stress. Our results show that salinity stress negatively affected tomato growth in terms of shoot length, fresh weight, and dry weight. In addition, we found that the inoculation of deep-sea *D. barathri* MT2.1^T^ clearly mitigated salt stress in tomatoes as these parameters were either equivalent or even higher than in non-inoculated tomatoes without stress (Figure 2a,b,d).

The RWC and MSI are two important physiological indices for evaluating salt stress effects [28,85]. Salt stress induces an excess of Na^+^ and Cl^−^ ions; consequently, it reduces plant water uptake ability, reflecting in a lower RWC [28,84]. Therefore, RWC is an indicator of plant water status used as an index for dehydration tolerance [86]. Our results show that RWC values of *D. profundi* MT2.2^T^-inoculated tomato seedlings were higher than in non-inoculated control under salt stress. These results suggested that this strain could help to maintain water status in tomatoes under salt stress. Similar results were reported in barley-inoculated with rare actinobacteria, *Curtobacterium flaccumfaciens* E108, and *Ensifer garamanticus* E110 under 4.4% NaCl concentration [56]. In contrast, tomato inoculated with neither *D. barathri* MT2.1^T^ or *D. nishinomiyaensis* DSM20448^T^ showed no significant difference with non-inoculated tomato plants under salt stress. Under salinity stress, the accumulation of salts in plant cells would damage the cell membrane integrity resulting in lower MSI [28]. Tomatoes inoculated with *D. nishinomiyaensis* DSM20448^T^ and *D. profundi* MT2.2^T^ showed higher MSI than non-inoculated tomatoes under 150 mM NaCl. Similar results were found in alfalfa plants inoculated with either *Hartmannibacter diazotrophicus* or *Pseudomonas* sp., which showed higher MSI than non-inoculated plants after 2 weeks at 10 dS m^−1^ and 20 dS m^−1^ salinity. The higher value of MSI indicates lower absorption of Na^+^ ions into the plant cells [13]. *D. barathri* MT2.1^T^ inoculated tomato showed lower MSI that was not significantly different from the non-inoculated tomato seedlings control. Although inoculation of deep-sea *D. barathri* MT2.1^T^ could not restore RWC and MSI value to normal status as in non-inoculated tomato plants without stress, salinity stress did not affect the overall growth of inoculated tomato as observed in shoot length, root length, and dry weight (Figure 2a,b,d).

Soluble sugars play an important role in maintaining the overall structure and development of plants to mitigate the negative effects of salt [87,88]. For example, sugars act as regulators of metabolism, photosynthesis, osmotic homeostasis, protein synthesis, and gene expression during various abiotic stress [88]. However, in the present study, the accumulation of TSS showed no positive effect on protecting tomato seedlings from salt stress. The concentration of TSS in tomato seedlings inoculated with *D. barathri* MT2.1^T^ and *D. profundi* MT2.2^T^ was lower than that in non-inoculated tomato plants under stress conditions. Although the inoculation of tomato seedlings with *D. nishinomiyaensis* DSM20448^T^ showed the highest TSS, no improvement in the overall growth performance as exemplified in the recorded dry weight compared with tomato seedlings inoculated with deep-sea *Dermacoccus* was found (Figure 2d). These results suggested that both *D. barathri* MT2.1^T^ and *D. profundi* MT2.2^T^ did not use TSS to mitigate salinity stress. Hu et al. [89] reported that the addition of a low concentration of exogenous glucose (0.1, 0.5 mM) under salt stress (200 mM NaCl) enhanced seed germination of wheat, increased chlorophyll content, dry weight, and proline accumulation, maintained ionic homeostasis, prevented water loss and stimulated antioxidant enzyme activity. Similarly, the addition of a lower concentration of exogenous glucose and sucrose promoted the growth of triticale (×*Triticosecale* Wittmack) seedlings under salt stress (100 mM NaCl) [90].

Proline is a compatible solute accumulated by plants under various stress conditions, such as salinity, water shortage, and extreme temperature [91,92]. Under salinity, plants accumulate proline in the cytoplasm to adjust osmotic pressure against salt-stress-induced water loss from cells [93]. In the present study, tomato inoculated with *D. nishinomiyaensis* DSM20448^T^ and *D. profundi* MT2.2^T^ increased proline content in tomato leaves. However, only tomato seedlings inoculated with *D. barathri* MT2.1^T^ showed better growth in all parameters (shoot length, root length, fresh and dry weight) than non-inoculated tomatoes under stress. Previously, studies on mungbean using *Bacillus cereus* Pb25 showed an increase in proline content in inoculated plants under salinity (NaCl 9 dS m^−1^) and increased fresh and dry root and shoot weight and yield compared to control [93]. On the contrary, proline content in tomatoes inoculated with *D. barathri* MT2.1^T^ was reduced. Although the inoculation of *D. barathri* MT2.1^T^ resulted in lower proline content in tomato leaves, the tomato seedlings showed better overall growth performance than non-inoculated tomatoes under stress and other treatments. In agreement with our results, Palaniyandi et al. [31] reported that tomatoes inoculated with *Streptomyces* sp. PGPA39 contained lower proline content than non-inoculated tomatoes but exhibited higher dry biomass and chlorophyll content under salinity stress (180 mM NaCl). Similarity, inoculation and co-inoculation of *Arthrobacter* strain MS-1 and MS-2 with symbiotic bacteria (*Sinorhizobium meliloti* strains R1 and R2) decreased proline content in leaves and improved growth rate, number of nodules, and salt tolerance in alfalfa under salinity stress (100 mM NaCl) [39]. Therefore, our results suggested that *D. barathri* MT2.1^T^ may use another alternative approach in combination with proline accumulation to alleviate salinity stress.

Salinity stress increases ROS in plant cells including H_2_O_2_ accumulation which affects several plant physiology in particular stomatal closure [33,94]. Stomatal closure was suggested to play role in plant adaptation during stress by preventing water loss through transpiration [94]. Chloroplast ultrastructure destruction, chlorophyll malfunction, and chlorophyll content reduction were also reported, which ultimately lowered or inhibited photosynthesis of plants [16]. Tomato seedlings inoculated with deep-sea actinobacteria showed higher chlorophyll content than control seedlings and demonstrated a positive effect on the growth and development of tomato seedlings in terms of shoot length, root length, and dry weight for *D. barathri* MT2.1^T^; shoot and root length for *D. profundi* MT2.2^T^. Similarly, *Streptomyces* sp. PGPA39-inoculated tomato showed higher total chlorophyll in leaves than non-inoculated tomato under both non-stress and salt stress (180 mM NaCl) conditions and improved plant dry biomass [31]. An increase in chlorophyll content was observed in mungbean (*Vigna radiata* L.) inoculated with *Bacillus cereus* Pb25 under 180 mM NaCl resulting in a higher fresh and dry root and shoot weight and yield compared to control plants [93]. In addition, chlorophyll content of maize inoculated with *Azotobacter* spp. C5 and C9 under salinity (5.85 g NaCl kg^−1^) was increased and resulted in a positive effect on maize growth in terms of root and shoot length, root and shoot dry weight [95]. In contrast, inoculation of terrestrial *D. nishinomiyaensis* DSM20448^T^ resulted in decreased chlorophyll content in leaves of tomato seedlings. Salinity stress is known to induce Fe-related deficiency, including chlorosis in plants [30]. Microbial siderophores have high specificity toward Fe and play a significant role in Fe availability for plants. Iron is required for enzymes responsible for chlorophyll synthesis. Interestingly, *D. barathri* MT2.1^T^ produced the highest hydroxamate siderophore, approximately two times higher than other *Dermacoccus* strains. This observation corresponded well with the recorded total chlorophyll content in tomato inoculated with *D. barathri* MT2.1^T^ that was two times higher than other treatments. Indeed, siderophore-producing bacteria have been shown to confer salt tolerance in several plant species, e.g., wheat [71,96,97]. These previous studies and the present study results suggest the role of siderophores in mitigating salinity stress in plants.

Hydrogen peroxide (H_2_O_2_) is a ROS indicator of oxidative stress in plants under various stress, including salinity. The overproduction of ROS causes cell death via various pathways, such as lipid peroxidation, protein oxidation, nucleic acid damage, and programmed cell death [98]. An increase in antioxidant enzyme activities indicates high oxidative stress in plants [99]. In the present study, H_2_O_2_ in leaves of tomatoes inoculated with *D. barathri* MT2.1^T^ and *D. profundi* MT2.2^T^ was lower than that in non-inoculated tomatoes under salinity (Figure 3d). Deep-sea *Dermacoccus* might enhance H_2_O_2_ scavenging enzymes, such as catalase (CAT) and peroxidase (POD), which help maintain H_2_O_2_ homeostasis and lead to plant growth under salt stress. Previously, inoculation of *B. cereus* Pb25 in mungbean under 9 dS m^−1^ NaCl increased CAT and POD enzymes resulted in a reduction of H_2_O_2_ level [93]. Later, soybean inoculated with *Pseudomonas* sp. strain AK-1 and *Bacillus* sp. strain SJ-5 under 100 mM NaCl showed lower catalase activity than non-inoculated plants. However, the peroxidase enzyme observed in soybean plants inoculated with SJ-5 strain was higher than in non-inoculated plants [98]. On the contrary, *D. nishinomiyaensis* DSM20448^T^-inoculated tomato showed a similar level of H_2_O_2_ to that found in non-inoculated tomato plants under salt stress. This observation suggests that tomatoes inoculated with *D. nishinomiyaensis* DSM20448^T^ and non-inoculated tomatoes were encountering similar stress. Our results showed that H_2_O_2_ production was reduced by the inoculation of tomato seedlings with the deep-sea *Dermacoccus*. This data supported the view that these deep-sea *Dermacoccus* have the potential to alleviate salt stress in tomato seedlings by promoting H_2_O_2_ reduction.

### 3.3. Biosafety Tests for Actinobacteria

#### 3.3.1. Pathogenicity Bioassay Based on *Caenorhabditis elegans*

Biosafety of bacterial strains intended to be used as PGPB is of paramount importance before releasing the plant biostimulant [100]. *Caenorhabditis elegans* is an alternative model to study infectious diseases in higher organisms, including humans [65,101,102]. *C. elegans* is suitable for use as a model because 36% of its 19,000 proteins in the genome match with those in humans [101]. In the bioassay, we evaluated the virulence of *Dermacoccus* on the number of adults, juveniles, eggs, and the death of nematodes (*C. elegans*). Nematodes fed with a non-pathogenic strain (*E. coli* OP50) showed the highest growth and reproduction and the lowest death rate at 144 h. In contrast, when fed with a pathogenic strain (*P. aeruginosa* PA14), nematodes died after 96 h, which indicated that these strains were harmful to *C. elegans*. All *Dermacuccus* strains, except *D. barathri* MT2.1^T^, showed a negative effect on the survival and fertility of *C. elegans*. Nematodes fed with *D. barathri* MT2.1^T^ showed the lowest severity compared to other *Dermacoccus*. This strain showed a death rate of only 1.8% compared to *E. coli* OP50 (1.6%). *D. nishinomiyaensis* DSM20448^T^ (23%) and *D. profundi* MT2.2^T^ (11%) showed a higher death rate than *D. barathri* MT2.1^T^. However, the death rate of all *Dermacoccus* strains was lower than that of the pathogenic *P. aeruginosa* PA14. This observation suggested that *D. barathri* MT2.1^T^ is considered to be safer than other *Dermacoccus* strains.

#### 3.3.2. *Escherichia coli* MC4100 Sensitivity

A sensitivity test of *E. coli* MC4100 viability was performed to assess harmful products release from marine actinobacteria, which were used as plant growth-promoting actinobacteria on soil microbial communities. We have shown that the survival rate of *E. coli* MC4100 exposed to supernatants from two marine actinobacteria, *D. barathri* MT2.1^T^ and *D. profundi* MT2.2^T^ was similar to the rate of non-pathogenic *P. putida* KT2440. Our results suggested that no deleterious effects on microbial communities occur when secondary metabolites produced by these marine *Dermacoccus* are released in soils.

#### 3.3.3. Ecotoxicity Test in Earthworms (*Eisenia foetida*)

Earthworms are important organisms in the soil and are a key indicator of ecosystem health [103]. Assessment of the risk that PGPB may have on earthworms is necessary. Therefore, we investigated the potential effects of *Dermacoccus* on earthworm (*E. foetida*) development. Earthworms fed with chickpea supplemented with *P. putida* KT2440, *D. nishinomiyaensis* DSM20448^T^, and *D. barathri* MT2.1^T^ showed a similar growth pattern regarding total weight and length compared to control chickpea. A similar number of eggs was recorded in all treatments with bacteria, being the lowest number observed in the negative control (2% NaCl). These results suggest that our *Dermacoccus* strains were safe for soil organisms, at least in earthworms.

#### 3.3.4. *Daphnia magna* Toxicity Bioassay

*Daphnia magna* is a planktonic crustacean used as a marker for toxicity screening of bacterial substances in the aquatic ecosystem [65]. Assay reports as the EC50 value (the concentration of bacterial supernatants that can kill 50% of *D. magna*). The EC50 value of supernatants from *Dermacoccus* strains was higher than EC50 from the pathogenic strain *B. cepacia* CC-A174. This observation supports the safety of using *Dermacoccus* strains in the aquatic ecosystem. Low EC50 value indicated a high degree of toxicity on *D. magna* as exemplified in *B. cepacia* CC-A174 [65]. These results in conjunction with those observed in *C. elegans* would suggest that *D. barathri* MT2.1^T^ is safe enough for its use as a plant biostimulant for their protection against salinity. However, additional biosafety tests are needed to recommend the safe use of *D. profundi* MT2.2^T^.

## 4. Materials and Methods

### 4.1. Bacterial Strains

Two deep-sea actinobacteria isolated from Mariana Trench sediments in the western Pacific Ocean: *D. barathri* MT2.1^T^ [64], and *D. profundi* MT2.2^T^ [64] and type species of the genus, *D. nishinomiyaensis* DSM20448^T^, were included in this study for plant experiment. *Burkholderia cepacia* CC-A174, *Escherichia coli* OP50, *E. coli* MC4100, *Pseudomonas aeruginosa* PA14, and *P. putida* KT2440 were used for biosafety tests. All bacterial strains were routinely cultivated on tryptic soy agar (TSA) (Difco BBL, Sparks, MD, USA) and tryptic soy broth (TSB) (Difco BBL, Sparks, MD, USA) at room temperature.

### 4.2. Plant Growth-Promoting Properties In Vitro

- Indole-3-acetic acid (IAA)

The IAA concentration was determined by the standard colorimetric method described by Lasudee et al. [68] with some modifications. Actinobacteria (1 loop full) were grown in glucose yeast extract broth (glucose 10 g, yeast extract 10 g, and distilled water 1000 mL) supplemented with L-tryptophan (2 mg mL^−1^) on a shaker at 110 rpm for 7 days in the dark. The following treatments were investigated: (1) control (No NaCl), (2) 150 mM NaCl, (3) 300 mM NaCl and (4) 450 mM NaCl. After 7 days, the supernatant was collected by centrifugation at 11,000 rpm for 5 min. The supernatant (1 mL) was mixed with 2 mL of Salkowski’s reagent [104] and incubated at room temperature for 30 min. IAA production was detected spectrophotometrically by measuring the absorbance at 530 nm. The quantity of IAA production was determined from a standard curve.

- Siderophores

Siderophore production was determined using chrome azurol S (CAS) agar [105]. Each actinobacterium (1 loop full) was cultured on TSA at room temperature for 3 days. Actinobacteria were inoculated on CAS agar and incubated at 28 °C for 7 days in the dark. The yellow or orange halo zone around the agar plugs on a blue background on CAS agar indicates siderophore production. Quantitative analysis of siderophore production was performed by growing each actinobacterium in King’s B broth (proteose peptone#3 10 g, glycerol 10 mL, K_2_HPO_4_ 1.5 g, MgSO_4_ 1.5 g, and distilled water 1000 mL) on a shaker at 110 rpm for 7 days. Hydroxamate type and catecholate type siderophores were detected by ferric perchlorate assay [106] and Arnow assay [107], respectively.

- Phosphate Solubilization

Screening of phosphate solubilizing activity was determined on Pikovskaya’s agar (PVK) supplemented with 0.5% (*w*/*v*) tri-calcium phosphate [108]. Each actinobacterium (1 loop full) was cultured on TSA at room temperature for 3 days. Actinobacteria were inoculated on PVK agar and incubated at 28 °C for 7 days. A clear zone of tri-calcium phosphate solubilization around the agar plugs indicates phosphate solubilization. For quantifying the released phosphorus, actinobacteria were grown in Pikovskaya’s broth (glucose 10 g, tri-calcium phosphate 5 g, NaCl 0.2 g, KCl 0.2 g, MgSO_4_ 0.1 g, MnSO_4_ 0.0025 g, Fe_2_(SO_4_)_3_ 0.0025 g, (NH_4_)_2_SO_4_ 0.5 g, and distilled water 1000 mL) supplemented with 0.5% (*w*/*v*) tri-calcium phosphate on a shaker at 110 rpm at room temperature for 7 days. The supernatant was collected to determine phosphorus released in culture broth using a colorimetric method [109].

- 1-Aminocyclopropane-1-Carboxylate (ACC) Deaminase Activity

ACC deaminase activity was detected based on the method of Palaniyandi et al. [31]. All actinobacteria were grown on a minimal medium [110] without nitrogen source (negative control), minimal medium supplemented with ammonium sulfate (positive control), and minimal medium supplemented with ACC at a final concentration of 3 mmol L^−1^. The incubation of all agar plates was performed at 30 °C for 7 days. Strains able to grow on ACC as nitrogen source exhibit a similar growth to those cultured in (NH_4_)_2_SO_4_ containing medium (positive ACC deaminase), whereas strains that could not utilize ACC show similar growth to those cultured in a medium without a nitrogen source (negative ACC deaminase).

### 4.3. Preparation of Inoculum for Plant Experiments

All actinobacteria were grown in TSB at room temperature on a shaker at 110 rpm for 48–72 h. Cells were collected by centrifugation at 5000 rpm for 15 min. Then, cells were resuspended in 0.5X M9 buffer and adjusted to 10^8^–10^9^ CFU/mL, as described by Narváez-Reinaldo et al. [111].

### 4.4. Enhancement of Tomato Seedlings Growth without Salt Stress

This experiment is designed to test the growth-promoting ability of *Dermacoccus* on tomato seedlings without salt stress. Seedlings of 1-month-old tomato (*Solanum lycopersicum*, Raf tomato) were purchased from SaliPlant S.L. specialist grower (Granada, Spain), were planted in the pot, containing a mixture of 50% (*v*/*v*) plant substrate and 50% (*v*/*v*) vermiculite. The following treatments were prepared: (1) control (non-inoculated) 3 mL M9 buffer were added, (2) *D. barathri* MT2.1^T^, (3) *D. profundi* MT2.2^T^, and (4) *D. nishinomiyaensis* DSM20448^T^ suspension. Each actinobacterial suspension (3 mL) contained 10^8^–10^9^ CFU/mL in 0.5X M9 buffer was added per seedling. Tomato seedlings were watered regularly until the end of the experiment. The following parameters were performed at days 0 and 26: shoot length, root length, fresh weight, and dry weight [112].

### 4.5. Enhancement of Tomato Seedlings Growth under Salt Stress

All *Dermacoccus* strains that promoted tomato growth without salt stress were selected to test their protective effect on tomato seedlings under salt stress. Seedlings of 1-month-old tomato (*Solanum lycopersicum*, Raf tomato) from SaliPlant S.L. specialist grower (Granada, Spain) were planted in trays size 2.5 × 2.5 × 7 cm and were regularly watered for 7 days. The pots were incubated in a growth room at a constant relative humidity (50–60%). The room was lit with a 12-h day/night cycle and gradual dimming/brightening of the light to simulate dawn and dusk. The day cycle consisted of 200 μmol photons·m^−2^·s^−1^, and the dawn-dusk cycle consisted of 150 μmol photons·m^−2^·s^−1^. The temperature was programmed to change from 18 to 20 °C for the night cycle to 20–25 °C in the diurnal [57]. Salt stress condition was applied to the seedlings by adding 150 mM NaCl solution, and this was considered as day 0 of the experiment. Electrical conductivity (EC1:5) was increased from 0.329 ± 0.02 dS m^−1^ in absence of NaCl to 1.158 ± 0.04 dS m^−1^ for samples containing 150 mM NaCl. The following treatments were investigated: (1) control (non-inoculated with stress) 0.5X M9 buffer was added and (2) actinobacterial suspension (10^8^–10^9^ CFU/mL in 0.5X M9 buffer). The actinobacterial suspensions prepared as described in 4.3 were applied at the rate of 2 mL/seedling by means of pouring. In addition, non-stressed treatment without bacterial inoculation was also carried out for comparison purposes. The following parameters were investigated after 14 days of inoculation: fresh weight (FW), root length, shoot length. Turgid weight (TW) was recorded after soaking the roots in distilled water for 48 h in the dark at room temperature. Dry weight (DW) was recorded after drying plants in the oven at 60 °C until constant weight [112]. Relative water content (RWC) was estimated according to the formula {RWC (%) = [(FW−DW)/(TW−DW) × 100]} [113]. Membrane stability index (MSI) was determined according to Asharf et al. [28]. Briefly, leaves of tomato (0.1 g) were washed in running tap water, washed in 10 mL distilled water, and heated at 40 °C for 30 min. The electrical conductivity was recorded (C1). The same sample was boiled in a water bath at 100 °C for 10 min, and electrical conductivity was recorded (C2). MSI was calculated as MSI = [1 − (C1/C2)] × 100.

#### Biochemical Analysis

Proline content in plants was assayed using a colorimetric method according to Bates et al. [113]. Tomato leaves (250 mg) were crushed by pestle and mortar with 3 mL 95% ethanol. Supernatants were collected by centrifugation at 1500 rpm for 5 min. The supernatant of each sample (200 µL) was mixed with 300 µL distilled water and 2 mL of ninhydrin reagent. The mixture was boiled at 100 °C for 1 h and the reaction was stopped on an ice bath. After that, 6 mL of toluene were added and vortexed for 20 s. The top organic layer was measured spectrophotometrically at 520 nm. Proline content was determined from the standard curve.

Chlorophyll content was determined according to Arnon [114]. Leaf samples (100 mg; W) were ground with 2.5 mL acetone (V), then centrifuged at 5000 rpm for 10 min. The optical density of supernatants was measured at 480, 663, and 645 nm by spectrophotometry. Chlorophyll content was calculated according to the following equation:

Total chlorophyll (mg g^−1^) = 20.2 × OD645 + 8.02 × OD663 × V/(1000 × W)

Total soluble sugar content (TSS) was estimated according to Dubois et al. [115]. Leaf samples (10 mg) were crushed with 1 mL 80% ethanol using mortar and pestle and incubated in a water bath at 75 °C for 15 min. Supernatants were collected by centrifugation at 12,000 rpm at 4 °C for 15 min; 250 µL of each supernatant sample was mixed with 250 µL of 80% ethanol, 2.5 mL of concentrated sulfuric acid, and 0.5 mL of 5% (*w*/*v*) phenol. The mixtures were kept for 20 min at room temperature; then, the optical density was measured at 520 nm with a spectrophotometer. TSS content was determined from the standard curve.

The concentration of hydrogen peroxide (H_2_O_2_) was examined according to Velikova et al. [116]. Leaf samples (100 mg) were ground with 3 mL 0.1% (*w*/*v*) of trichloroacetic acid in an ice bath and centrifuged at 10,000 rpm at 4 °C for 15 min. Each supernatant was mixed with 0.5 mL of 10 mM potassium phosphate buffer (pH 7.0) and 1 mL of 1 M potassium iodine. The optical density was measured at 390 nm. The quantity of H_2_O_2_ was determined from the standard curve.

### 4.6. Biosafety Tests for Actinobacteria

#### 4.6.1. Pathogenicity Bioassay Based on *Caenorhabditis elegans*

This bioassay was performed as described by Vilchez et al. [65]. Each actinobacteria culture broth was dropped in a T-shaped line (100 µL) on five potatoes dextrose agar (PDA) plates. Plates were kept at 30 °C for 3 h to dry. Plates were maintained at 22 °C for 24 h before use. Five juvenile nematodes at larval stage L3-L4 were added to each plate. Plates were incubated at 22 °C, and the number of adults, juveniles, deposited eggs, and dead nematodes were counted every 24 h for 7 days. *Pseudomonas aeruginosa* PA14 was used as a positive control of pathogenic strains and *Escherichia coli* OP50 as a positive control to estimate the natural growth and death rate of nematodes.

#### 4.6.2. *Escherichia coli* MC4100 Sensitivity

This bioassay was performed following the method described by Vilchez et al. [65]. Cell suspension of *E. coli* MC4100 in M9 buffer (10^8^–10^9^ CFU/mL) (5 mL) was mixed with 1 mL filtered sterile supernatant of a tested strain of actinobacteria. The mixture was incubated at room temperature for 1 h, then serially diluted and plated on TSA to estimate the number of surviving *E. coli* MC4100 (CFU/mL). *E. coli* suspension mixed with *Pseudomonas putida* KT2440 supernatant was used as a non-pathogenic strain and *P. aeruginosa* PA14 as a pathogenic strain [102,117].

#### 4.6.3. Ecotoxicity Test in Earthworms (*Eisenia foetida*)

Earthworm reproduction test using *E. foetida* was performed according to the Organisation for Economic Co-operation and Development (OCDE) Test Guideline No. 222 [118,119] with slight modifications [65]. Actinobacteria were cultured in TSB at 30 °C for 72 h. Actinobacterial cells were collected by centrifugation, mixed with 20 mL of 1 M trehalose, and freeze-dried. Ten of same generation earthworms (at least 1 month old and about 5.5–6 cm long) were put in pots filled with 0.5 L of a mixture of vegetal substrate and sphagnum peat (3:1 *w/w*) and kept at pH 6.7–7.4, 70% relative humidity and 20–25 °C for 28 days. The earthworms were fed with 5 g chickpea per week as a control, 4.5 g chickpea mixed with 0.5 g *B. cepacia* CC-A174 as a pathogenic strain, and *P. putida* KT2440 as a non-pathogenic strain. For testing ecotoxicity, 0.5 g dry powder of each actinobacterium was mixed with 4.5 g chickpea and fed to earthworms. As a control for the earthworm death rate, NaCl (2% *w/w*) was added to the substrate mixture as a dry powder [57]. Length, weight, the number of ootheca, and the number of juveniles were recorded at 7, 14, 21, and 28 days and expressed as an average value of ten earthworms.

#### 4.6.4. *Daphnia magna* Toxicity Bioassay

The toxicity of actinobacterial supernatants to *D. magna* was tested with DaphToxKitFR (Microbiotests BE) [120] accordingly to ISO6341 and OECD Test Guideline no. 211 [121]. The ephippia were placed in standard fresh water and incubated at 20 °C for 72 h under continuous illumination. Twenty newly hatched daphnia were transferred to a multiwell microplate system with cell-free supernatant (10 mL/well; 5 daphnias/well). Cell-free supernatant from each actinobacterium was diluted in standard freshwater according to the manufacturer’s instructions. *B. cepacia* CC-A174 was used as a pathogenic strain and *P. putida* KT2440 as a non-pathogenic strain. Immobile cells were counted at 24 and 48 h.

### 4.7. Data Analysis

The in vitro and in vivo plant growth-promoting experiments were performed in triplicate and presented as mean values and standard deviations (SD). The data were analyzed using a one-way analysis of variance (ANOVA) and Duncan’s multiple range tests. The statistical analysis was performed using SPSS (version 17.0) at *p* < 0.05. Significantly different mean values were indicated with different letters.

## 5. Conclusions

In conclusion, our results clearly show that deep-sea, *D. barathri* MT2.1^T^ could promote the growth of tomato (*Solanum lycopersicum*) seedlings under salinity stress. Adverse effects of salt stress in tomato seedlings were reduced as observed in various growth parameters: shoot and root length, fresh and dry weight. These deep-sea *Dermacoccus* may reduce the oxidative stress in tomatoes under salt stress via hydrogen peroxide reduction, possibly by the production of H_2_O_2_ scavenging enzymes. Biosafety assay also suggested the safety of some deep-sea *Dermacoccus* strains in aquatic and terrestrial ecosystems. The different results observed in the biosafety level of the various studied strains would point to the need to include this type of assays for the various *Dermacoccus* species. This study provides the first supporting evidence on the potential of deep-sea actinobacteria to promote the growth of plants under salinity stress.

## Figures and Tables

**Table 1:** Indole-3-acetic acid production (µg mL^−1^) of actinobacteria under various sodium chloride concentrations.

Isolate	0 mM NaCl + L-Tryptophan	150 mM NaCl + L-Tryptophan	300 mM NaCl + L-Tryptophan	450 mM NaCl + L-Tryptophan
DSM20448^T^	16.64 ^a^ ± 9.59	9.39 ^a^ ± 5.47	89.82 ^b^ ± 12.15	101.01 ^b^ ± 8.61
MT2.1^T^	6.18 ^b^ ± 0.82	8.98 ^b^ ± 0.82	9.32 ^b^ ± 1.22	8.03 ^b^ ± 4.49
MT2.2^T^	12.50 ^a,b^ ± 10.61	7.73 ^a^ ± 0.73	21.25 ^b^ ± 3.12	19.76 ^b^ ± 4.48

Data represent the mean values of three replicates ± SD. Different letters (a,b) indicate a significant difference in IAA production in each NaCl concentration according to Duncan at *p* < 0.05.

**Table 2:** Siderophore production and phosphate solubilization of actinobacteria.

Isolate	Siderophore Production			Phosphate Solubilization		
	Yellow/Orange Zone on CAS Agar	Hydroxamate (µmol mL^−1^)	Catecholate (µmol mL^−1^)	Clear Zone on PVK Agar	P Released in PVK Broth (µg mL^−1^)	pH
DSM20448^T^	+	121.25 ^a^ ± 26.52	22.63 ^a^ ± 3.20	+	46.47 ^a^ ± 2.92	4.78 ^a^ ± 0.25
MT2.1^T^	+	240.00 ^b^ ± 21.21	10.18 ^c^ ± 4.29	+	169.27 ^b^ ± 3.11	4.62 ^a^ ± 0.04
MT2.2^T^	+	129.38 ^a^ ± 27.40	12.10 ^c^ ± 3.45	+	165.47 ^b^ ± 7.35	4.66 ^a^ ± 0.06

+: Positive results. Data represent the mean values of three replicates ± SD. Different letters (a,b) indicate a significant difference in siderophore production and phosphate solubilization among strains according to Duncan at *p* < 0.05.

## Data Availability

Data sharing does not apply to this article as no datasets were generated or analyzed during the current study.

## Supplementary Materials

The following are available online at https://www.mdpi.com/article/10.3390/plants10081687/s1, Figure S1: Growth of tomato seedlings inoculated with *Dermacoccus* under 150 mM NaCl. (a) Non-inoculated tomato without salt stress; (b) Non-inoculated tomato with salt stress; (c) *D. nishinomiyaensis* DSM20448^T^-inoculated tomato seedling; (d) *D. barathri* MT2.1^T^-inoculated tomato seedling; (e) *D. profundi* MT2.2^T^ -inoculated tomato seedling; Figure S2: ACC deaminase activity of *Dermacoccus* strains. (a) *Dermacoccus* grown on minimal medium supplemented with (NH_4_)_2_SO_4_; (b) *Dermacoccus* grown on minimal medium supplemented with ACC; (c) *Dermacoccus* grown on minimal medium.
