# Supplementary figures and images for: Deep-Sea Actinobacteria Mitigate Salinity Stress in Tomato Seedlings and Their Biosafety Testing
Source: Plants (Basel). 2021 Aug 17;10(8):1687. doi: 10.3390/plants10081687 (PMC8401925; doi:10.3390/plants10081687)

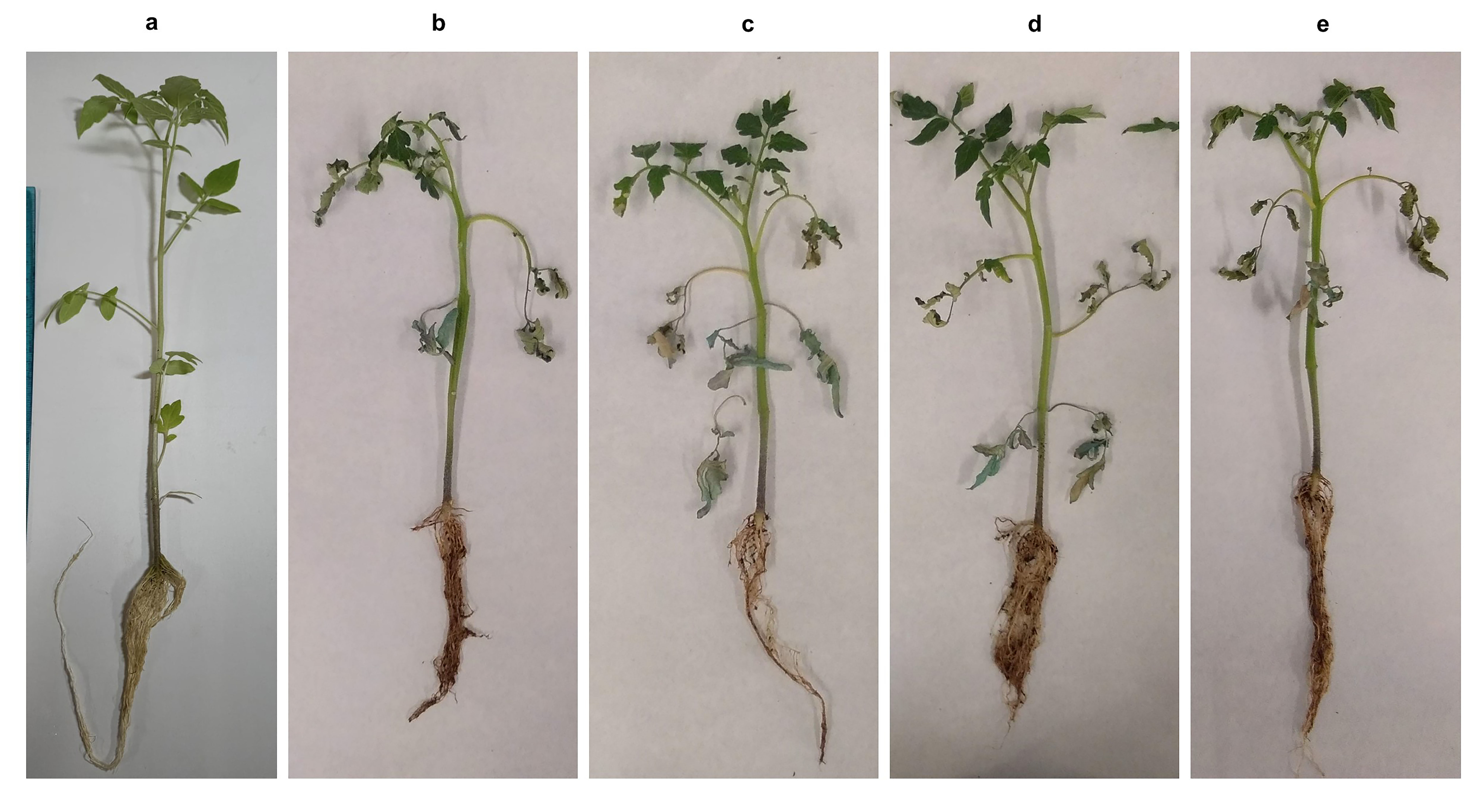

Supplement: Supplementary file 1 [file plants-10-01687-s001.zip › Supplementary Figure S1.jpg]

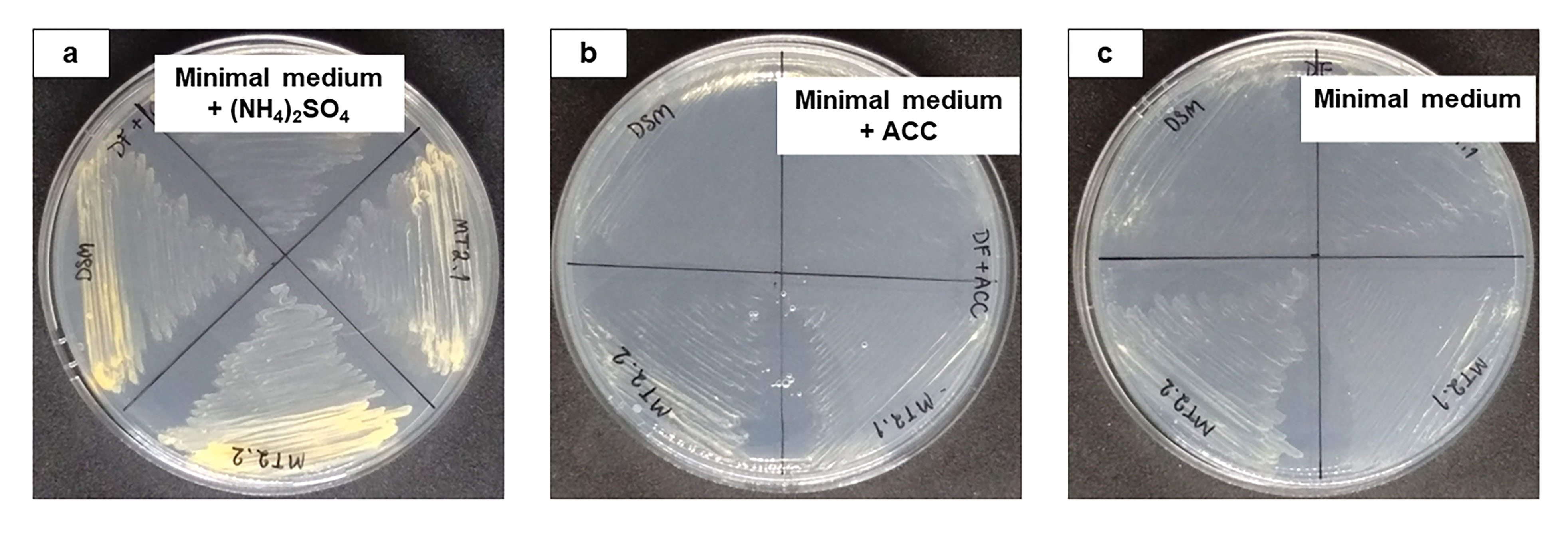

Supplement: Supplementary file 1 [file plants-10-01687-s001.zip › Supplementary Figure S2.jpg]
